# Modelling variable dropout in randomised controlled trials with longitudinal outcomes: application to the MAGNETIC study

**DOI:** 10.1186/s13063-016-1342-0

**Published:** 2016-04-28

**Authors:** Ruwanthi Kolamunnage-Dona, Colin Powell, Paula Ruth Williamson

**Affiliations:** MRC North West Hub for Trials Methodology Research, Department of Biostatistics, Institute of Translational Medicine, University of Liverpool, Liverpool, L69 3GA UK; Department of Child Health, Institute of Molecular and Experimental Medicine, Cardiff University School of Medicine, Heath Park, Cardiff, CF14 4XW UK; Department of General Paediatrics, Children’s Hospital for Wales, Heath Park, Cardiff, CF14 4XW UK

**Keywords:** Longitudinal outcome, Dropout process, Joint modelling, Competing risks

## Abstract

**Background:**

Clinical trials with longitudinally measured outcomes are often plagued by missing data due to patients withdrawing or dropping out from the trial before completing the measurement schedule. The reasons for dropout are sometimes clearly known and recorded during the trial, but in many instances these reasons are unknown or unclear. Often such reasons for dropout are non-ignorable. However, the standard methods for analysing longitudinal outcome data assume that missingness is non-informative and ignore the reasons for dropout, which could result in a biased comparison between the treatment groups.

**Methods:**

In this article, as a post hoc analysis, we explore the impact of informative dropout due to competing reasons on the evaluation of treatment effect in the MAGNETIC trial, the largest randomised placebo-controlled study to date comparing the addition of nebulised magnesium sulphate to standard treatment in acute severe asthma in children. We jointly model longitudinal outcome and informative dropout process to incorporate the information regarding the reasons for dropout by treatment group.

**Results:**

The effect of nebulised magnesium sulphate compared with standard treatment is evaluated more accurately using a joint longitudinal-competing risk model by taking account of such complexities. The corresponding estimates indicate that the rate of dropout due to good prognosis is about twice as high in the magnesium group compared with standard treatment.

**Conclusions:**

We emphasise the importance of identifying reasons for dropout and undertaking an appropriate statistical analysis accounting for such dropout. The joint modelling approach accounting for competing reasons for dropout is proposed as a general approach for evaluating the sensitivity of conclusions to assumptions regarding missing data in clinical trials with longitudinal outcomes.

**Trial registration:**

EudraCT number 2007-006227-12. Registration date 18 Mar 2008.

**Electronic supplementary material:**

The online version of this article (doi:10.1186/s13063-016-1342-0) contains supplementary material, which is available to authorized users.

## Background

Although the reasons for all participant dropouts in a trial may not be known, some might be related to the unobserved study outcome. Dropout may be caused by adverse reactions or a lack of effectiveness of the treatment, or it may be affected by the concurrent health status of the patient; hence the dropout is often informative or non-ignorable [1, 2].

Therefore, missing data due to study dropouts are a potential source of bias when analysing clinical trials, and it is important to evaluate the effect of informative dropout on the robustness of the study conclusions. Trial statisticians often carry out a sensitivity analysis as a feasible approach for this purpose (e.g., [3, 4]), and this has also been recommended by the Europe Medicines Agency Committee for Medicinal Products for Human Use. A recent review of the handling of missing longitudinal outcome data in clinical trials revealed that 36 % studies failed to account for reasons for dropout and carried out just a complete case analysis [5].

In this article, we explore the impact of informative dropout due to competing reasons on the evaluation of the effect of nebulised magnesium sulphate in the MAGNEsium Trial in Children (MAGNETIC; Current Controlled Trials identifier ISRCTN81456894). MAGNETIC is the largest randomised, double-blind, placebo-controlled study to date comparing the addition of nebulised magnesium sulphate to standard treatment in acute severe asthma in children. It was funded by the National Institute for Health Research (NIHR) Health Technology Assessment programme. MAGNETIC enrolled children from 30 hospitals in the United Kingdom. They were aged between 2 and 16 years, had severe acute asthma and did not respond to standard inhaled treatment. The complete eligibility criteria are given in a 2013 article in *Health Technology Assessment* [6]. Children were randomly allocated (1:1) to isotonic magnesium sulphate (MgSO_4_) or placebo (isotonic saline) on three occasions at 20-minute intervals. The main objective of the trial was to determine whether the use of nebulised MgSO_4_, when given as an adjunct to standard therapy for 60 minutes, results in a clinical improvement compared with standard treatment alone. The severity of asthma was assessed using the validated Yung Asthma Severity Score (ASS). ASS was recorded at randomisation and then at 20 minutes (after first nebuliser treatment); 40 minutes (after second nebuliser treatment); 60 minutes (after third nebuliser treatment); and at further follow-up points 120, 180 and 240 minutes post-randomisation. The Yung ASS ranges from 0 to 9. A low ASS indicates an improved outcome. In the study, 252 children were randomised to the magnesium group and 256 to the placebo group. Further details of the trial, including how sample size was determined, randomisation, blinding, and primary and secondary outcomes were published in 2013 in *Health Technology Assessment* [6] and *Lancet Respiratory Medicine* [7]. As with most longitudinal studies, the MAGNETIC trial also encountered patient dropout during both the treatment and follow-up phases. The reasons for dropout were sometimes clearly known and recorded during the trial; however, in many instances these reasons were unknown or unclear.

We jointly model longitudinal ASS and dropout process due to competing risks in the MAGNETIC trial to incorporate the information regarding the reasons for dropout in both the magnesium and placebo groups. Competing risks occur when an event (dropout in this case) could happen due to several causes, and one cause excludes the event of interest due to other causes. In the MAGNETIC trial, competing causes of dropout included good prognosis, poor prognosis, and unrelated and unknown reasons. Competing risks are usually analysed using risk regression models such as cause-specific Cox proportional hazards or cumulative incidence models (e.g., [8–10]). More recently, several authors have extended these models to include longitudinal profiles through joint longitudinal-competing risks modelling (e.g., [11–13]). Although such joint analyses are statistically more efficient for evaluating treatment accounting for the competing dropout process, to our knowledge these approaches have been underused in the clinical trials literature, potentially due to a lack of research that demonstrates the implementation of this approach in practice.

## Methods

### Dropout definitions

Dropout is defined when sequences of longitudinal measurements in some patients terminate prematurely. In clinical trials, patients who withdrew from the study or were lost to follow-up were considered as dropouts. The time at which they withdrew or were lost to follow-up is defined as the time to dropout. Following the terminology of Diggle and Kenward [14], we define dropout mechanisms as

1. *Completely random dropout* (CRD): The dropout and longitudinal processes are independent.
2. *Random dropout* (RD): The dropout process depends on the observed longitudinal measurements (i.e., those preceding dropout).
3. *Informative dropout* (ID): The dropout process depends on the unobserved longitudinal measurements (i.e., those that would have been observed if the patient had not dropped out).

According to the Rubin [1] terminology for missingness (rather than “dropout”), CRD, RD and ID mechanisms are termed *missing completely at random*, *missing at random* and *missing not at random*, respectively. Motivated by the reasons for withdrawals recorded in the MAGNETIC trial, we can define the dropout process within four possible categories:

1. *Case 1*: good prognosis
2. Case 2: poor prognosis
3. *Case 3*: unknown (or unclear) reasons
4. *Case 4*: reasons unlikely to be related to the patient’s concurrent health status

Case 1 is observed if a patient is withdrawn from the trial due to improving conditions (e.g., discharged from the hospital as the patient was ready to continue treatment at home). Case 2 is observed if a patient is withdrawn from the trial due to worsening conditions (e.g., patient needed more intensive treatment at an intensive care unit or the occurrence of an adverse event). Both cases 1 and 2 include dropout reasons that are directly related to the patient’s concurrent health status; hence both cases depend on the unobserved longitudinal measurements and should be classified under the ID mechanism. Any dropout that occurred due to an inconclusive reason (case 3) can be considered to include a mixture of CRD, RD and ID mechanisms. Dropout reasons may be identified that are not likely to be related to a patient’s concurrent health status (e.g., protocol deviation, trained assessor not available to take the measurement). In the MAGNETIC study, potential reasons falling under this fourth category were determined by the chief investigator (CP), with that assessment undertaken blinded to the treatment allocation.

### Standard joint longitudinal and event-time model

In the standard joint model, dropout is treated under ID mechanism but allows dropout only as a single event-time process, so the differential reasons for dropout cannot be considered. The model is defined as shown below. The longitudinal outcome is assumed to follow a Gaussian linear model:

1 $$ {Y}_t=\boldsymbol{X}{\boldsymbol{\beta}}_1+{W}_1(t)+{Z}_t $$

where *W*_1_(*t*) is a latent zero-mean Gaussian process, measurement error *Z*_*t*_ follows a zero-mean Gaussian process with variance *σ*^2^, that error is independent of the latent process *W*_1_(*t*), and *X* allows treatment allocation and any other covariates. Conditional on latent effects, *W*_1_(*t*), a single event-time (dropout) process, follows a semi-parametric proportional hazards model:

2 $$ \lambda \left(t\Big|\boldsymbol{X},\;{W}_2(t)\right)={\lambda}_0(t) exp\left\{\boldsymbol{X}{\boldsymbol{\beta}}_2+{W}_2(t)\right\} $$

where *W*_2_(*t*) is a second latent zero-mean Gaussian process. Within standard formulation, we assume *W*_1_(*t*) = *U*_0_ + *U*_1_*t* in conjunction with a proportionality assumption *W*_2_(*t*) = *γW*_1_(*t*), where *U*_0_ and *U*_1_ are individual random intercept and random slope terms, respectively [15]. The link between *W*_1_ and *W*_2_ describes the association between the longitudinal outcome and dropout process, and *γ* denotes the strength of this association. ***β***_1_ in model (1) estimates the covariate effects adjusted for the dropout process. In model (2), if the treatment is fitted as a binary covariate *X*, taking a value 1 if active treatment and 0 if standard, then the corresponding HR ${e}^{\beta_2}$ estimates the risk of dropout in the treatment group compared with standard (or control) adjusted for the temporal variation of the longitudinal outcome. The model parameters can be estimated by maximising the joint likelihood of the observed data via the expectation-maximisation (EM) algorithm (see [15] for more details).

### Joint longitudinal-competing risks model

The standard joint model with a single mode of dropout fails to account for the differential effect of treatments on the reasons for dropout. To account for the informative dropout due to competing risks, following the approach of Williamson et al. [13], we assume each competing reason for dropout follows a semi-parametric, cause-specific proportional hazards sub-model. Dropout due to reasons related to the *l*^th^ cause is defined by

3 $$ {\lambda}^{(l)}\left(t\Big|\boldsymbol{X},\;{W}_1(t)\right)={\lambda}_0^{(l)}(t) exp\left\{\boldsymbol{X}{{\boldsymbol{\beta}}_2}^{\left(\boldsymbol{l}\right)}+{\gamma}^{(l)}{W}_1(t)\right\},\;l=1,\;2,\dots,\;\mathrm{K} $$

where *W*_1_(*t*) = *U*_0_ + *U*_1_*t* is defined with latent intercept *U*_0_ and slope *U*_1_. *γ*^(*l*)^ indicates the level of association between the longitudinal outcome and *l*^th^ competing dropout process. K is the total number of dropout causes (e.g., for MAGNETIC, K = 4). If the treatment is fitted as a binary covariate, taking a value 1 if active treatment and 0 if standard, then the corresponding HR ${e}^{{\beta_2}^{(l)}}$ estimates the relative risk of dropout in the treatment group compared with standard due to reasons related to the *l*^th^ cause adjusted for the temporal variation in the longitudinal outcome. The longitudinal outcome is defined by model (1), and we have fitted the following linear mixed effects model for the longitudinal ASS:

4 $$ {Y}_t={\beta}_0+{\beta}_1X+{\beta}_2\mathrm{t}+{U}_0+{U}_1t+{Z}_t $$

where *U*_0_ ~ *N*(0, *σ*_0_^2^) and *U*_1_ ~ *N*(0, *σ*_1_^2^) are random intercept and random slope, respectively, assuming Corr (*U*_0_, *U*_1_) = *ρ*, while the measurement error *Z*_*t*_ is defined as in model (1). *X* is the treatment covariate taking value 1 if magnesium and 0 if placebo. The predicted ASS for magnesium (*X* = 1) at time *t* is given by ${\widehat{\beta}}_0+{\widehat{\beta}}_1+{\widehat{\beta}}_2\mathrm{t}$, and for placebo (*X* = 0) it is ${\widehat{\beta}}_0+{\widehat{\beta}}_2\mathrm{t}$. Hence, *β*_1_ is the estimated difference in ASS in magnesium against placebo at any time *t* over follow-up. In the joint competing risks model, *β*_1_ is adjusted for the association between *Y*_*t*_ (longitudinal ASS) and the competing dropout process.

The model parameters are estimated by maximising the joint likelihood via the EM algorithm [13]. However, as K increases, the estimation could become more computationally intensive in a standard computing environment due to the high number of association parameters. Further details of the likelihood-based estimation process are given by Williamson et al. [13]. Bootstrap sampling with replacement is used to estimate 95 % CIs [16].

### Methods for dealing with complications in MAGNETIC dropout process

The most efficient approach would be to consider all possible competing causes of dropout in a competing risks joint model. However, in the MAGNETIC trial, the number of patients who dropped out due to poor prognosis (case 2) was low in both treatment groups (see Table 1), leading to a lack of convergence of the longitudinal-competing risks model. Therefore, we propose two separate scenarios. In the first scenario, we randomly assign case 3 equally between cases 1 and 2 for each treatment group (a more conservative scenario). The second is the worst-case scenario; here we assume all unknown reasons for dropout (case 3) were related to poor prognosis and assign as case 2 for each treatment group. So, we allow dropout due to unknown reasons under the ID mechanism. Any case 4 dropout is assumed to have occurred under the CRD mechanism and thus is independently censored at the time of dropout. Those who have completed the follow-up schedule are also independently censored at the final follow-up of 240 minutes post-randomisation. In the above-described settings, we explore informative dropout due to two competing risks (K = 2): good prognosis and poor prognosis.

**Table 1:** Summary of dropout reasons by treatment at the final follow-up

Status	Magnesium, *n* (%)	Placebo, *n* (%)	Total, *n* (%)
Baseline ASS assessment completed	248 (49.4)	254 (50.6)	502
Completed follow-up	185 (74.6)	217 (85.4)	402 (80.1)
Overall dropout	63 (25.4)	37 (14.6)	100 (19.9)
Case 1: Due to good prognosis	10 (15.9)	5 (13.5)	15 (15.0)
Case 2: Due to poor prognosis	1 (1.6)	3 (8.1)	4 (4.0)
Case 3: Due to unknown or unclear reasons	11 (17.4)	9 (24.3)	20 (20.0)
Case 4: Unlikely to be related to the patient’s concurrent health status	41 (65.1)	20 (54.1)	61 (61.0)

*ASS* Asthma Severity Score

### Ethical considerations

The MAGNETIC study was approved by the U.K. National Health Service Multicentre Research Ethics Committee (MREC 07/H1010/101) and by the U.K. National Health Service Medicines for Children Research Network. Written informed consent was obtained from a parent or guardian of each child who was enrolled in the study.

## Results and discussion

**Fig. 1:**
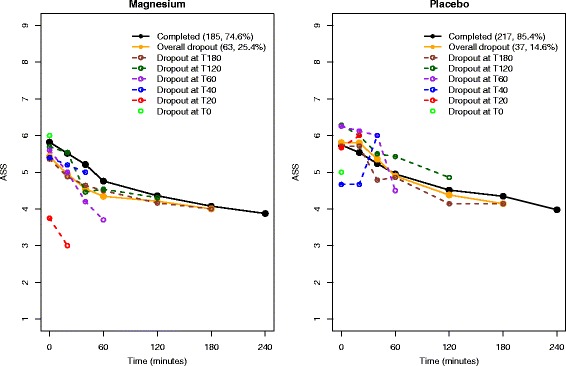
Longitudinal mean Asthma Severity Score (ASS) profiles for groups according to whether they completed follow-up or dropped out

The MAGNETIC trial data includes 248 children (49 %) in the magnesium group and 254 children (51 %) in the placebo group. The Consolidated Standards of Reporting Trials (CONSORT) flow diagram and checklist are presented for this analysis (see Additional files 1 and 2). Table 1 presents the summary of the dropout process in MAGNETIC. A higher overall dropout rate is observed in the magnesium group than in the placebo arm, with some suggestion that the reasons for dropout differ between groups. Figure 1 shows the mean longitudinal ASS profiles for those who have completed the follow-up schedule and for those who have dropped out at each assessment point, together with the overall dropout profile. Dropouts in the magnesium group occurred with lower ASS due to patients improving and being discharged before the end of follow-up, whereas early dropouts in the placebo group occurred with higher ASS as a result of patients worsening and needing more intense treatment. Although the graphical summaries of ASS dropout trajectories indicated that most children were clinically well and ready to be discharged at the time of withdrawal from the trial in the magnesium group [6], this claim is yet to be justified on the basis of a formal statistical analysis.

### Standard analyses ignoring competing dropout reasons

The results from two standard analyses are shown in Table 2. The complete case analysis is the most common method and is usually undertaken before the sensitivity analyses. The complete case analysis excludes patients who dropped out of the study, assuming that patients who dropped out are the same as those who completed the trial; in other words, it assumes a CRD mechanism for all dropouts. To illustrate the method, we have fitted the linear mixed effect model (4) for the longitudinal ASS for those who had completed the follow-up schedule at all *t* at 0, 20, 40, 60, 120, 180 and 240 minutes (*n* = 402 patients) (Table 1). The model has estimated a non-significant treatment effect (${\widehat{\beta}}_1=$ −0.086, 95 % CI −0.297 to 0.125), implying that MgSO_4_ shows no improvement of ASS against placebo over time. The primary analysis of the MAGNETIC trial was an analysis of covariance to test the hypothesis of no difference between the two treatment arms at *t* = 60 minutes, and a difference of −0.25 points (95 % CI −0.48 to −0.02) was noted, which lies above the minimum clinically important difference of 0.5 points [6, 7]. The current model takes the variation of ASS over the entire follow-up and estimates a difference of −0.086. However, the assumed CRD mechanism is evidently incorrect for the MAGNETIC trial, as some of the reasons for dropout were clearly related to the patient’s concurrent health status, and some were unknown (Table 1).

**Table 2:** Estimates (95 % CI) from the complete case and standard joint modelling^a^ analyses

Model	Longitudinal outcome	Dropout	
	*β*_1_ (95 % CI)	HR (95 % CI)	*γ* (95 % CI)
Complete case analysis (*n* = 402)	−0.086 (−0.297 to 0.125)	–	–
Standard joint model (*n* = 502, overall dropouts = 100)	−0.193 (−0.381 to −0.010)	1.869 (1.238 to 2.772)	−0.211 (−0.442 to −0.017)

^a^95 % bias-corrected percentile CIs are obtained from 1000 bootstrap resamples

The standard joint longitudinal ASS and dropout model assumes all dropouts occur under the ID mechanism (as a single event-time process) and is fitted by

$$ {Y}_t={\beta}_0+{\beta}_1X+{\beta}_2\mathrm{t}+{W}_1(t)+{Z}_t $$

$$ \lambda \left(t\Big|X,\;{W}_1(t)\right)={\lambda}_0(t) exp\left\{{\beta}_2X+\gamma {W}_1(t)\right\} $$

where *X* is the treatment covariate taking value 1 if magnesium and 0 if placebo; *t* can take values in 0, 20, 40, 60, 120, 180 and 240 minutes before dropout; and *W*_1_(*t*) = *U*_0_ + *U*_1_*t*. This analysis includes the entire sample of 502 patients, and the model estimates a statistically significant improvement in ASS for MgSO_4_ over placebo (*β*_1_ = −0.193, 95 % CI −0.381 to −0.010). The estimated association parameter *γ* = −0.211 (95 % CI −0.442 to −0.017) implies that dropout is associated with lower ASS, and the corresponding HR = 1.832 (95 % CI 1.238 to 2.772) indicates that the risk of dropout is significantly higher in the magnesium group. As low ASS indicates better asthma control, the estimated *γ* and HR parameters indicate that dropouts in the magnesium group had significantly low ASSs and that MgSO_4_ significantly reduces ASS. However, the standard joint analysis has ignored the differential effects of magnesium and placebo on reasons for dropout, and also that some reasons for dropout are unknown. Therefore, the validity of the above-mentioned claim is questionable.

### Analysis of informative dropout due to competing reasons

**Fig. 2:**
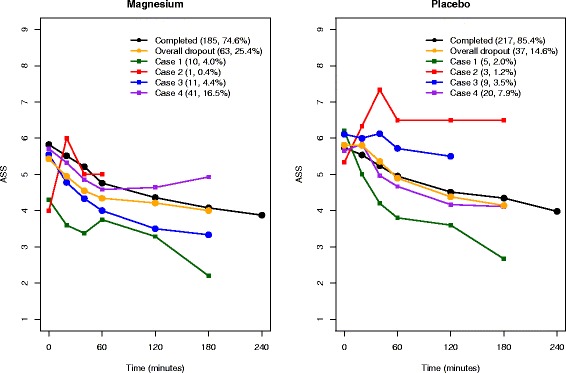
Longitudinal mean Asthma Severity Score (ASS) profiles for competing dropout processes, together with the mean ASS profile for those who completed

Figure 2 shows mean longitudinal ASS dropout profiles for the four cases, together with the mean profiles for those who have completed the follow-up schedule and the overall dropout profile. The mean profile of the three patients who dropped out due to poor prognosis (case 2) from the placebo group shows the highest ASS. The mean dropout profiles from case 4 remain similar to the profiles of those completing follow-up in both treatment groups. Therefore, the CRD mechanism seems a reasonable assumption for the case 4 group. However, mean dropout profiles for case 3 are clearly positioned on opposite sides from those completing for the magnesium and placebo groups; for the magnesium group, it is below (lower ASS, better prognosis) the profile for those who completed, whereas for the placebo group, it is above (higher ASS, worse prognosis). Case 3 should be considered under the ID mechanism in any analysis. Ignoring this situation could induce bias when estimating the treatment effect.

**Fig. 3:**
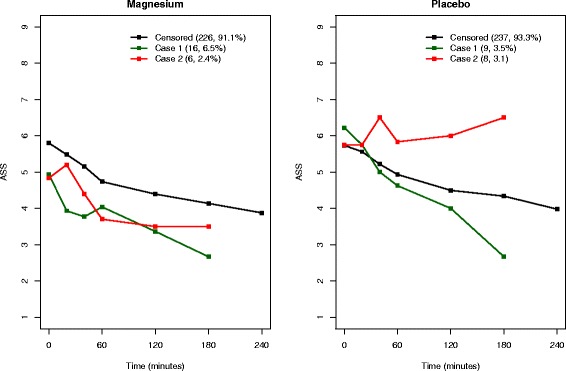
Scenario 1 (conservative): mean longitudinal Asthma Severity Score (ASS) profiles

**Fig. 4:**
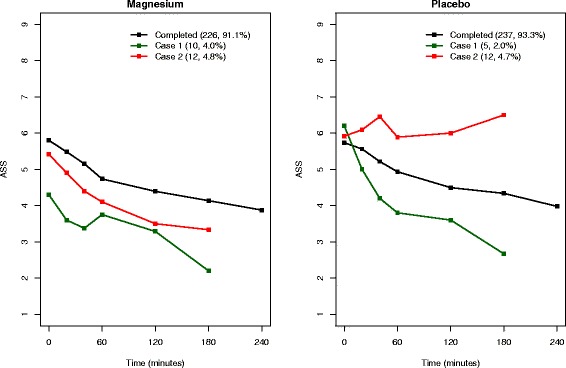
Scenario 2 (worst): mean longitudinal Asthma Severity Score (ASS) profiles

As discussed in the Methods section, we have re-defined cases 1 and 2 under the two scenarios. For scenario 1, of the 11 patients in case 3 in the magnesium group, 6 are assigned as case 1 and 5 as case 2 randomly, and of the 9 patients in placebo group, 4 are assigned as case 1 and 5 as case 2. Figures 3 and 4 show the corresponding mean ASS profiles for re-defined cases 1 and 2 under scenarios 1 and 2, respectively. For each scenario, we fit the joint ASS and competing risks model as follows:

$$ {Y}_t={\beta}_0+{\beta}_1X+{\beta}_2\mathrm{t}+{W}_1(t)+{Z}_t $$

$$ {\lambda}^{(1)}\left(t\Big|X,\;{W}_1(t)\right)={\lambda}_0^{(1)}(t) exp\left\{{\beta_2}^{(1)}X+{\gamma}^{(1)}{W}_1(t)\right\} $$

$$ {\lambda}^{(2)}\left(t\Big|X,\;{W}_1(t)\right)={\lambda}_0^{(2)}(t) exp\left\{{\beta_2}^{(2)}X+{\gamma}^{(2)}{W}_1(t)\right\} $$

where *X*, *t* and *W*_1_(*t*) are defined as above. The second and third components of the model are related to *l* = 1 and 2 in model (3) and allow reasons for dropout due to good and poor prognosis, respectively. These analyses include the entire sample of 502 patients, and the corresponding estimates are given in Table 3. Scenario 1 has been performed for a number of different random allocations to include ten patients each in cases 1 and 2, and all such allocations resulted in fairly similar estimates. The model estimates corresponding to the above-mentioned random allocation are presented in Table 3, and this model included 25 patients in case 1 and 14 in case 2. In scenario 2, case 1 includes 15 patients and case 2 includes 24 patients.

**Table 3:** Estimates (95 % CI^a^) from the joint longitudinal-competing risks model for scenario 1 (conservative) and scenario 2 (worst)

Scenario	Longitudinal outcome	Dropout due to good prognosis		Dropout due to poor prognosis	
	*β*_1_ (95 % CI)	HR^(1)^ (95 % CI)	*γ* ^(1)^ (95 % CI)	HR^(2)^ (95 % CI)	*γ* ^(2)^ (95 % CI)
1 (*n* = 502 case 1: 25 case 2: 14)	−0.165 (−0.336 to 0.011)	1.915 (0.820 to 3.507)	−0.768 (−1.340 to −0.299)	0.801 (0.122 to 1.872)	0.200 (−0.436 to 0.715)
2 (*n* = 502 case 1: 15 case 2: 24)	−0.163 (−0.363 to 0.010)	2.125 (0.845 to 3.904)	−1.389 (−2.021 to −0.938)	1.069 (0.350 to 2.148)	0.159 (−0.361 to 0.588)

^a^95 % bias-corrected percentile CIs are obtained from 1000 bootstrap resamples

Once the reasons for dropout were taken into account, the estimated improvement with respect to ASS for MgSO_4_ over placebo by the standard joint model (*β*_1_ = −0.193) was reduced by about 15 % in both scenarios (−0.163 and −0.165, respectively) and both estimates of *β*_1_ became non-significant; however, the change in absolute terms (0.03 and 0.028, respectively) was small and the estimated treatment effects (−0.163 and −0.165, respectively) remained above the minimum clinically important difference of 0.5 points [6, 7]. We expect this reduction in *β*_1_ estimates, given the differential ASS profiles between magnesium and placebo for the two competing dropout reasons, as shown in Figs. 3 and 4, which is not taken into account in the standard joint model.

For scenario 1, the estimated association parameter *γ*^(1)^ = −0.768 (95 % CI −1.340 to −0.299) implies that dropout due to good prognosis is strongly associated with low ASS; however, the HR, ${e}^{{\beta_2}^{(1)}}$ = 1.915 (95 % CI 0.820 to 3.507) implies that such dropout is not significantly higher in the magnesium group than that in the placebo group. *γ*^(2)^ = 0.200 (95 % CI −0.436 to 0.715) and the HR, ${e}^{{\beta_2}^{(2)}}$ = 0.801 (95 % CI 0.122 to 1.872), imply that dropout due to poor prognosis is not evident in the magnesium group. The estimates from the worst case scenario (scenario 2) are also indicated as the same; however, the association parameter *γ*^(1)^ = −1.389 (95 % CI −2.021 to −0.938) implies a much stronger association between ASS and risk of dropout due to good prognosis. In both scenarios, the corresponding HR estimates (1.915 in scenario 1 and 2.125 in scenario 2) indicate that the rate of dropout due to good prognosis is about twice as high in the magnesium group as in the placebo group. Further, for both scenarios, positive estimates of *γ*^(2)^ imply that patients with higher ASS have increased risk of dropout due to poor prognosis, and the negative estimates of *γ*^(1)^ imply that patients with low ASS have increased risk of dropout due to good prognosis. Therefore, the above analysis supports the assertion that most children in the magnesium group were clinically well at the time of withdrawal from the trial and that the estimated association parameters and corresponding HRs reveal the true impact of MgSO_4_ on ASS.

## Conclusions

Clinical trialists often perform sensitivity analyses to study the robustness of the estimated treatment effect to missing data. However, most sensitivity analyses are based on simple imputation methods (such as last observation carried forward or mean substitution [5]) and usually assume a CRD mechanism; hence they may fail when the reason for dropout is unknown or unclear and informative. According to a recent review [5], MAGNETIC is the first clinical trial that in which researchers have reported fitting the standard joint model in a sensitivity analysis to account for informative dropout [6]. Such statistically more efficient methodologies are currently underused in clinical trials analyses due to a lack of awareness resulting from limited examples of their application in practice. In this article, we have reported the dropout process in the MAGNETIC trial in more detail and demonstrated the use of a more efficient statistical methodology within the joint modelling framework to deal with the complex dropout process in the trial. Although the proposed methods were based on MAGNETIC trial data, they are generalisable across other longitudinal studies. We have programmed in R language to estimate the model parameters; however, a variety of software with alternative estimation procedures are available for this class of models, including the Bayesian approaches (e.g., SAS [SAS Institute, Cary, NC, USA], C, WinBUGS). The R code is available from the corresponding author on request.

We emphasise the importance of identifying reasons for dropout and undertaking statistically efficient analysis accounting for such dropout. The joint modelling approaches accounting for competing reasons for dropout is proposed as a general approach for evaluating the sensitivity of conclusions to assumptions regarding missing data in clinical trials with longitudinal outcomes.

## Additional files

Additional file 1: CONSORT diagram for MAGNETIC trial. CONSORT diagram of the analysis undertaken for this article using the MAGNETIC trial data. (DOCX 40 kb) Additional file 2: CONSORT checklist. CONSORT checklist corresponding to the analysis undertaken for this article using the MAGNETIC trial data. (DOCX 47 kb)

## Abbreviations

ASS: Asthma Severity Score
CONSORT: Consolidated Standards of Reporting Trials
CRD: completely random dropout
EM: expectation-maximisation
ID: informative dropout
MAGNETIC: MAGNEsium Trial in Children
NIHR: National Institute for Health Research
RD: random dropout
