# Supplementary material for: Modelling variable dropout in randomised controlled trials with longitudinal outcomes: application to the MAGNETIC study
Source: Trials. 2016 Apr 28;17:222. doi: 10.1186/s13063-016-1342-0 (PMC4849065; doi:10.1186/s13063-016-1342-0)
Supplement: Additional file 1: — CONSORT diagram for MAGNETIC trial. CONSORT diagram of the analysis undertaken for this article using the MAGNETIC trial data. (DOCX 40 kb) [file 13063_2016_1342_MOESM1_ESM.docx]

**Allocated to and RECEIVED** **PLACEBO (n=256)**

**Allocated to and RECEIVED** **MAGNESIUM (n=252)**

**Discontinued intervention with no further data collection (n=63)**

Due to good prognosis (n=10)

Due to poor prognosis (n=1)

Due to unknown/unclear reasons (n=52)

**ANALYSED (N=248)**

Excluded from analysis as one item of ASS at baseline was not recorded (n= 4)

**ANALYSED (N=254)**

Excluded from analysis as one item of ASS at baseline was not recorded (n=2)

**Discontinued intervention with no further data collection (n=37)**

Due to good prognosis (n=5)

Due to poor prognosis (n=3)

Due to unknown/unclear reasons (n=29)

**Analysis**

**Follow-up**

**RANDOMISED (N=508)**

**Enrolment**

**Allocation**
